# A peripheral signature of Alzheimer’s disease featuring microbiota-gut-brain axis markers

**DOI:** 10.1186/s13195-023-01218-5

**Published:** 2023-05-31

**Authors:** Moira Marizzoni, Peppino Mirabelli, Elisa Mombelli, Luigi Coppola, Cristina Festari, Nicola Lopizzo, Delia Luongo, Monica Mazzelli, Daniele Naviglio, Jean-Louis Blouin, Marc Abramowicz, Marco Salvatore, Michela Pievani, Annamaria Cattaneo, Giovanni B. Frisoni

**Affiliations:** 1 Laboratory of Biological Psychiatry, IRCCS Istituto Centro San Giovanni Di Dio Fatebenefratelli, Brescia, Italy; 2 Laboratory of Neuroimaging and Alzheimer’s Epidemiology, IRCCS Istituto Centro San Giovanni Di Dio Fatebenefratelli, Brescia, Italy; 3 IRCCS SYNLAB SDN, Naples, Italy; 4 Department of Pharmacological and Biomolecular Sciences, University of Milan, Milan, Italy; 5 Istituto Di Biostrutture E Bioimmagini (I.B.B.) - CNR, Naples, Italy; 6 Dip.to Di Scienze Chimiche, Università Degli Studi Di Napoli - Federico II, Naples, Italy; 7 Genetic Medicine Division, University Hospitals and University of Geneva, Geneva, Switzerland; 8 Memory Clinic and LANVIE - Laboratory of Neuroimaging of Aging, University Hospitals and University of Geneva, Geneva, Switzerland

**Keywords:** Cognitive impairment, Alzheimer’s disease, Gut microbiota, Microbiota-gut-brain axis, Lipopolysaccharide, Endothelial dysfunction

## Abstract

**Background:**

Increasing evidence links the gut microbiota (GM) to Alzheimer’s disease (AD) but the mechanisms through which gut bacteria influence the brain are still unclear. This study tests the hypothesis that GM and mediators of the microbiota-gut-brain axis (MGBA) are associated with the amyloid cascade in sporadic AD.

**Methods:**

We included 34 patients with cognitive impairment due to AD (CI-AD), 37 patients with cognitive impairment not due to AD (CI-NAD), and 13 cognitively unimpaired persons (CU). We studied the following systems: (1) fecal GM, with 16S rRNA sequencing; (2) a panel of putative MGBA mediators in the blood including immune and endothelial markers as bacterial products (i.e., lipopolysaccharide, LPS), cell adhesion molecules (CAMs) indicative of endothelial dysfunction (VCAM-1, PECAM-1), vascular changes (P-, E-Selectin), and upregulated after infections (NCAM, ICAM-1), as well as pro- (IL1β, IL6, TNFα, IL18) and anti- (IL10) inflammatory cytokines; (3) the amyloid cascade with amyloid PET, plasma phosphorylated tau (pTau-181, for tau pathology), neurofilament light chain (NfL, for neurodegeneration), and global cognition measured using MMSE and ADAScog. We performed 3-group comparisons of markers in the 3 systems and calculated correlation matrices for the pooled group of CI-AD and CU as well as CI-NAD and CU. Patterns of associations based on Spearman’s rho were used to validate the study hypothesis.

**Results:**

CI-AD were characterized by (1) higher abundance of *Clostridia_UCG-014* and decreased abundance of *Moryella* and *Blautia* (*p* < .04); (2) elevated levels of LPS (*p* < .03), upregulation of CAMs, Il1β, IL6, and TNFα, and downregulation of IL10 (*p* < .05); (3) increased brain amyloid, plasma pTau-181, and NfL (*p* < 0.004) compared with the other groups. CI-NAD showed (1) higher abundance of *[Eubacterium] coprostanoligenes group and Collinsella* and decreased abundance of *Lachnospiraceae_ND3007_group*, *[Ruminococcus]_gnavus_group* and *Oscillibacter* (*p* < .03); (2) upregulation of PECAM-1 and TNFα (*p* < .03); (4) increased plasma levels of NfL (*p* < .02) compared with CU. Different GM genera were associated with immune and endothelial markers in both CI-NAD and CI-AD but these mediators were widely related to amyloid cascade markers only in CI-AD.

**Conclusions:**

Specific bacterial genera are associated with immune and endothelial MGBA mediators, and these are associated with amyloid cascade markers in sporadic AD. The physiological mechanisms linking the GM to the amyloid cascade should be further investigated to elucidate their potential therapeutic implications.

**Supplementary Information:**

The online version contains supplementary material available at 10.1186/s13195-023-01218-5.

## Background

The gut microbiota (GM) represents the most densely populated bacterial community colonizing the human body and covers a wide range of functions critical to several aspects of human health. The GM is a key driver of the innate immune system [1, 2] and is involved in the degradation of macronutrients and production of metabolites [3]. Intestinal bacteria and their products modulate endothelial cell function and, at the same time, intestinal epithelial cells influence immune responses and shape the microbial composition [4] and generate a barrier preventing the passage of antigens and bacteria from the gut into the bloodstream [5].

Alzheimer’s disease (AD) pathology is characterized by the extracellular accumulation of β-amyloid (A), thought to facilitate intracellular cortical deposition and spreading of hyper-phosphorylated tau (T) which in turn drives progressive neurodegeneration (N), ultimately leading to cognitive impairment (CI) [6]. Clinical and preclinical evidence supports GM involvement in promoting AD onset and progression. Individuals with AD dementia and in the AD preclinical state reported alterations in the GM composition compared with healthy controls [7–11], and amyloid deposition was associated with pro-inflammatory bacterial species in the gut and pro-inflammatory cytokines in the blood [12]. Preclinical studies showed that GM is necessary for brain amyloid deposition [13–15] and is associated with neurodegeneration [16–18]. While growing evidence suggests that GM may impact cognitive impairment via signaling molecules of the microbiota-gut-brain axis (MGBA) [19–21], few studies in humans have assessed their relationship with markers of the amyloid cascade (ATN scheme) and CI. Furthermore, to the best of our knowledge, the simultaneous evaluation of a large panel of immune and endothelial mediators and their association with GM in AD patients has not been described previously.

We hypothesized that the fecal microbial alterations of patients with CI due to AD are associated with a specific profile of immune and endothelial MGBA mediators in the blood and that the latter is linked to markers of the amyloid cascade. To evaluate the existence and specificity of such a peripheral signature of sporadic AD, we investigated the association of fecal bacterial genera, MGBA mediators, and amyloid cascade markers in cognitively unimpaired persons (CU), patients with CI not due to AD (CI-NAD) and patients with CI due to AD (CI-AD).

## Materials and methods

The present study shares several aspects with two previous papers focused on the evaluation of the role of GM in AD [12, 21]. Differently from those studies, here we applied a whole genome instead of a candidate approach for the GM characterization and we evaluated also tau pathology and neurodegeneration.

### Study participants

Participants were community-dwelling persons of 50 to 85 years of age recruited from a large Italian study on amyloid imaging in patients with cognitive complaints, the Incremental Diagnostic Value of [18F]-Florbetapir Amyloid Imaging [INDIA-FBP] study [22]. As previously reported, all participants underwent an extensive neuropsychological battery (Additional file 1) including global cognitive measures (Mini-Mental State Examination (MMSE) and the Alzheimer’s Disease Assessment Scale, cognitive portion (ADAS‐Cog)). The inclusion criteria for participants with normal cognition were at most one neuropsychological test score outside the normal range. CI was defined as (i) presence of cognitive complaints reported by patients or proxy or by the physician; (ii) absence of intracranial metabolic or psychiatric causes of cognitive impairments; (iii) presence of abnormal scores in ≥ 2 cognitive tests; and (iv) history of progression of cognitive symptoms. Amyloid positivity was used to classify participants into amyloid-positive (CI-AD) and amyloid-negative (CI-NAD and CU) and was defined as global [18F]-Florbetapir standardized uptake value ratio versus cerebellum (SUVR) higher than 1.11 [23]. The 85 subjects included in the present study were not under antibiotic nor anti-inflammatory treatment over the past 3 months and accepted to donate stools and blood. Venous blood samples were collected from all participants using 4-ml K3-ethylenediamine tetra-acetic acid (EDTA) vacutainer and centrifuged at 3400 g for 10 min at 4 °C within 2 h of collection to obtain plasma. Plasma samples were then aliquoted and stored at − 80 °C until testing. Stool samples were collected from subjects at their own home in a sterile plastic cup, stored at − 20 °C, and delivered to IRCCS Fatebenefratelli Institute in Brescia within the following 24 h, where they were stored at − 20◦C until their processing.

### Fecal bacterial composition

DNA was extracted from 180 to 200 mg of frozen stool using the QIAamp DNA Stool Mini Kit (Qiagen Retsch GmbH, Hannover, Germany) and according to the manufacturer’s instructions. Bead-beating homogenization by TissueLyser II (Qiagen Retsch GmbH, Hannover, Germany) was performed to mechanically disrupt fecal samples before DNA extraction. The samples were homogenized for 10 min at 30 Hz. DNA was quantified using a NanoDrop ND-1000 spectrophotometer, and then stored at + 4 °C until subsequent analyses. The regions V3 and V4 of the bacterial ribosomal RNA 16S gene were amplified and purified according to 16S Metagenomic Sequencing Library Preparation protocol by Illumina (Additional file 2). A paired-end read of 300 cycles per read was performed. The raw 16S data were processed using QIIME2 [24] (64 bit version 2021.4) run on a MacBook Pro with Intel CPU 12 × 2.6 GHz processors and 16 GB of RAM. Sequencing Illumina MiSeq data were already demultiplexed. Forward and reverse primers, reads containing ambiguous bases, or homopolymers greater than eight base pairs in length were removed. Moreover, we set a maximum number of expected errors equal to 2 and reads truncation if the quality score was less than 2. The DADA2 denoising process was applied with the default parameters. Alpha diversity, indicating the richness and abundance of ASVs within each individual, and beta diversity, showing the similarity or difference in microbiota composition between individuals, were calculated using the q2-diversity plugin after rarefaction of the feature table using the sample depth corresponding to the sample with the lowest read count. SILVA reference database (version 138) (https://www.arb-silva.de/), customized following the instructions on the dedicated tutorials and as previously reported (paper 16S), was used to infer the taxonomy of the amplicon sequence variants (ASV) at phylum and genus level. Absolute abundances at the genus and phylum levels were normalized by the total number of reads assigned in each sample. Genera assigned to the Archaea domain or found with relative abundance lower than 10^−2^, in less than 4 subjects across all samples were removed. A total of 139 ASVs were identified in the whole group after filtering. Alpha and beta diversity were calculated using the q2-diversity plugin and included Bray–Curtis and Jaccard indexes, observed ASVs, phylogenetic diversity, and Shannon and Pielou’s evenness indexes. The feature table was rarefied to the sample depth corresponding to the sample with the lowest read count.

### Microbiota-gut-brain axis mediators

A series of markers were selected in order to investigate several possible *immune and endothelial* mediators of the MGBA: the gut bacteria product LPS; soluble CAMs involved in AD and indicative of endothelial damage (sVCAM-1 and sPECAM-1 [25, 26]), vascular damage (sP- and sL-Selectin [27]) and upregulated in response to infection (sNCAM and sICAM-1 [28, 29]); inflammatory and anti-inflammatory cytokines reported to be altered in AD (IL1β, IL6, TNFα, IL18, IL10 [30]). CAMs were selected because of the key role of endothelial cells in enabling communication between visceral organs and the central nervous system, providing information about physiological conditions in the body, and adapting accordingly to maintain homeostasis [31, 32]. LPS was measured in plasma by ELISA (Pierce LAL Chromogenic Endotoxin Quantitation Kit, Thermo Fisher Scientific). sCAMs were measured using the LEGENDplex™ Human Adhesion Molecule Panel multiplex assay (Cat.#740,945, BioLegend). Data were analyzed with LEGENDplex™ Cloud-based Data Analysis Software (Dec 05, 2019, BioLegend). For cytokines expression level, total RNA was isolated using the PAXgene blood miRNA kit according to the manufacturer’s protocol (PreAnalytiX, Hombrechtikon, CHE), and candidate gene expression analyses were performed using real-time PCR. Each target gene was normalized to the expression of three reference genes, glyceraldehyde 3-phosphate dehydrogenase, beta-actin, and beta-2-microglobulin, using TaqMan Assays on a 384-well Real-Time PCR System (Biorad). The expression levels of each target gene were normalized to the geometric mean of all three reference genes, and the Pfaffl method was used to determine the relative target gene expression of each gene in patients as compared with controls.

### Amyloid cascade markers

[18F]-Florbetapir Amyloid PET was performed as previously reported [12, 21]. In addition, other markers altered during the pathological processes of AD were measured. Plasma concentrations of NFL (NF-Light immunoassay Advantage kit; Cat. N° 103,400), GFAP (GFAP Human Discovery Kit; Cat. N° 102,336), and p- tau 181 V2 Advantage Kit (p- tau181; Cat. No. 103714) were measured using the ultrasensitive Simoa SR-X instrument in accordance with the manufacturer’s recommended protocol. General cognition was assessed using MMSE and ADAScog.

### Statistical analysis

Statistical analyses were performed using GraphPad Prism (v 8.1.1) (GraphPad Software, San Diego, CA, USA), unless specified otherwise. The comparison among groups of descriptive statistics, GM products, endothelial and inflammatory markers were performed using ANOVA with Bonferroni correction for continuous Gaussian variables (or Kruskall-Wallis test with Dunn correction for non-Gaussian variables) and Chi-square test for categorical data. Two-group comparison (CI-NAD vs CU; CI-AD vs CU) in the abundance of ASVs grouped at phylum and genera levels were determined with Linear discriminant analysis effect size (LEfSe) [33], performed in the Galaxy platform (http://huttenhower.sph.harvard.edu/galaxy/) and visualized using GraphPad Prism. Beta and alpha diversity comparison analyses were performed in QIIME2 by applying the Kruskall-Wallis test and the permutational multivariate analysis of variance (PERMANOVA), respectively. Three-dimensional principal coordinate analysis (PCoA) was used to visualize beta diversity results. Associations of genera with MGBA mediators and of the latter with amyloid cascade markers were assessed with Spearman correlation, the nonparametric version of the Pearson correlation which reduces the influence of extreme values. The effect of age was calculated using the Partial and Semi-Partial (Part) Correlation (“ppcor”) R package (v 1.1) [34]. Significance was set at *p* < 0.05 (two-tailed) and, for hypothesis validation analyses, associations were selected if their Spearman’s rho value was > 0.4 in order to include only those with at least moderate association.

## Results

Demographic and clinical characteristics were as expected for this population (Table 1 and Additional file 3). First, we compared the markers of the physiological systems among the clinical groups to identify those associated with sporadic AD. Then, we assessed the association of genera with MGBA mediators and of the latter with amyloid cascade markers.

**Table 1:** Demographic and clinical features of study participants

	CU	CI-NAD	CI-AD	*P*-value	
				ANOVA ^b^	CI-NAD vs CI-AD ^c^
*N*	13	38	34		
Age (years)	69.6 (7.1)	69.8 (7.4)	70.8 (6.1)	.796	> .999
Female	7 (54%)	21 (55%)	16 (47%)	.775	.487
Education (years)	9.1 (5.2)	8.5 (3.9)	8.7 (4.5)	> .999	> .999
Body mass index^a^	25.0 (3.4)	25.3 (3.8)	24.9 (2.9)	> .999	> .999
APOEe4 carrier status^d^	2 (18%)	3 (9%)	20 (65%)	** < .001**	** < .001**
General cognition					
Mini-Mental State Examination	28.3 (1.2)	24.8 (3.9)	22.2 (5.1)	** < .001**	0.101
ADAScog	8.2 (3.3)	15.3 (9.5)	17.1 (7.6)	** < .001**	.330
Clinical stage					
Mild cognitive impairment	-	21 (55%)	22 (65%)		.415
Dementia	-	17 (45%)	12 (35%)		.415
Amyloid load (PET SUVr)	.95 (.07)	.92 (.09)	1.31 (.13)	** < .001**	** < .001**

Figure denotes mean (SD) and number (%)

Abbreviations: CU: cognitively unimpaired persons; CI-NAD: patients with cognitive impairment not due to AD; CI-AD: patients with cognitive impairment due to AD; ADAScog: Alzheimer's Disease Assessment Scale, cognitive subscale; PET: Positron emission tomography; SUVr: Standardized uptake value ratio

^a^ Weight/height^2^ and measured in kg/cm^2^

^b^ Statistical difference among the 3 groups by ANOVA, Kruskal–Wallis, or chi-squared test

^c^ *P* values of pairwise comparisons using Bonferroni correction

^d^ Missing data for 8 participants: 2 in CU, 4 in CI-NAD, and 3 in CI-AD

### Gut microbial communities

**Fig. 1:**
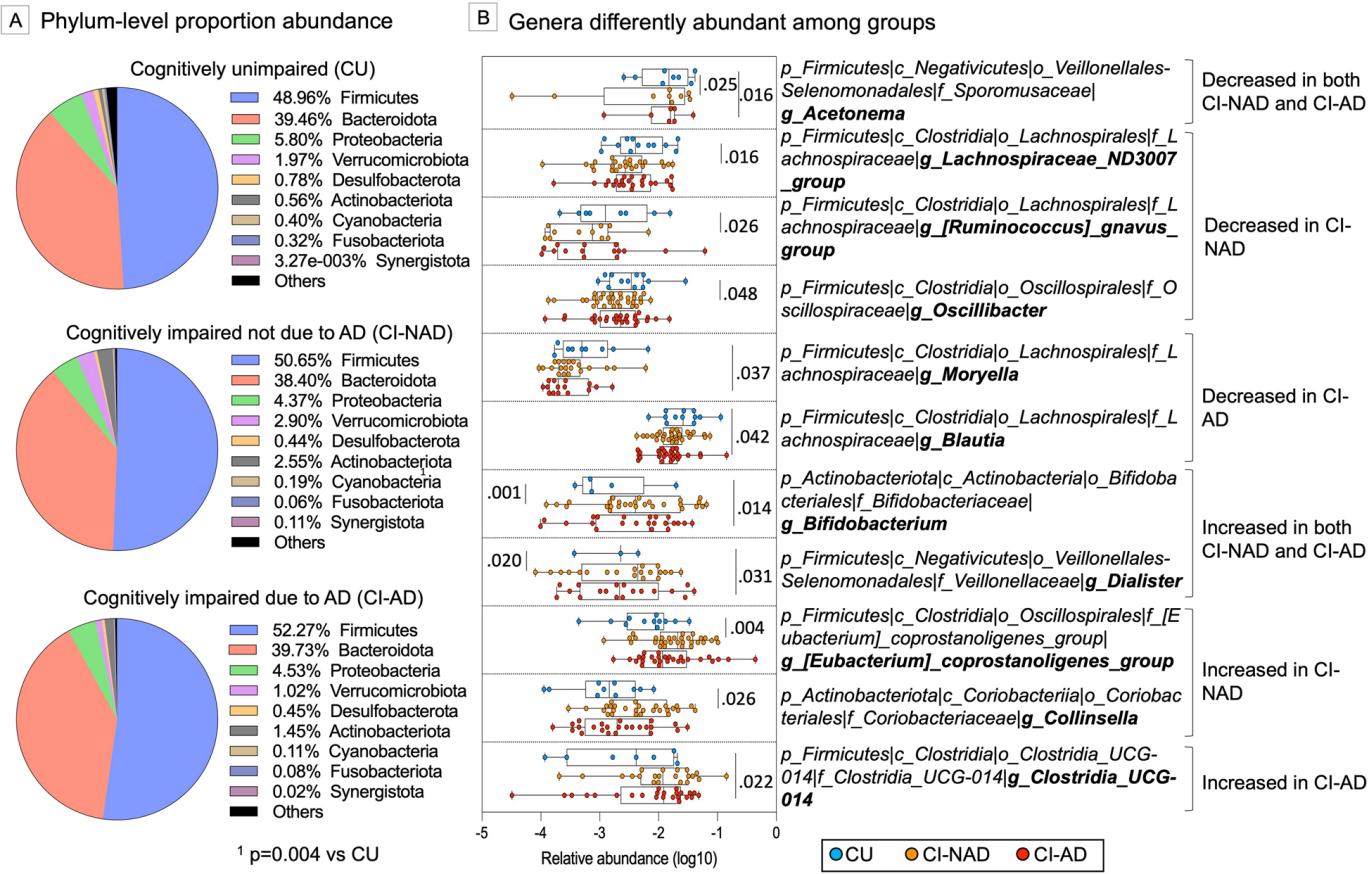
Gut microbial communities of study participants grouped at phylum (**A**) and genus (**B**) levels. *P*-values were calculated using the online LEfSe workflow on the Hutlab Galaxy platform. Log10 transformation of genera abundances was used to increase readability. Abbreviations: CU, cognitively unimpaired persons; CI-NAD, patients with cognitive impairment not due to AD; CI-AD, patients with cognitive impairment due to AD

The comparison of beta diversity metrics did not show any GM compositional difference among groups (PERMANOVA, Bray–Curtis, and Jaccard distances, *p* > 0.133; Additional file 4A-B). Similar results were obtained for alpha diversity where the groups were comparable for all the metrics considered (*p* > 0.176; Additional file 4C). Concerning phylum taxonomic comparison, a shift in the abundance of *Firmicutes* (+ 3.3% in CI-NAD and + 6.3% in CI-AD) and *Actinobacteria* (+ 78% in CI-NAD and + 61% in CI-AD) was observed in CI groups compared to CU (Fig. 1A). However, only the increase of *Actinobacteria* in CI-NAD was significant (*p* = 0.004). Genus comparison revealed that both CI groups reported a lower abundance of *Acetonema* and a higher abundance of *Bifidobacterium* and *Dialister* compared to CU (*p* < 0.031). CI-AD were characterized by decreased abundance of *Moryella* and *Blautia* and a higher abundance of *Clostridia_UCG-014* and (*p* < 0.042) while CI-NAD by decreased abundance of *Lachnospiraceae_ND3007_group*, *[Ruminococcus]_gnavus_group* and *Oscillibacter* as well as a higher abundance of *[Eubacterium] coprostanoligenes group and Collinsella,* and (*p* < 0.026).

### Immune and endothelial microbiota-gut-brain axis mediators

**Fig. 2:**
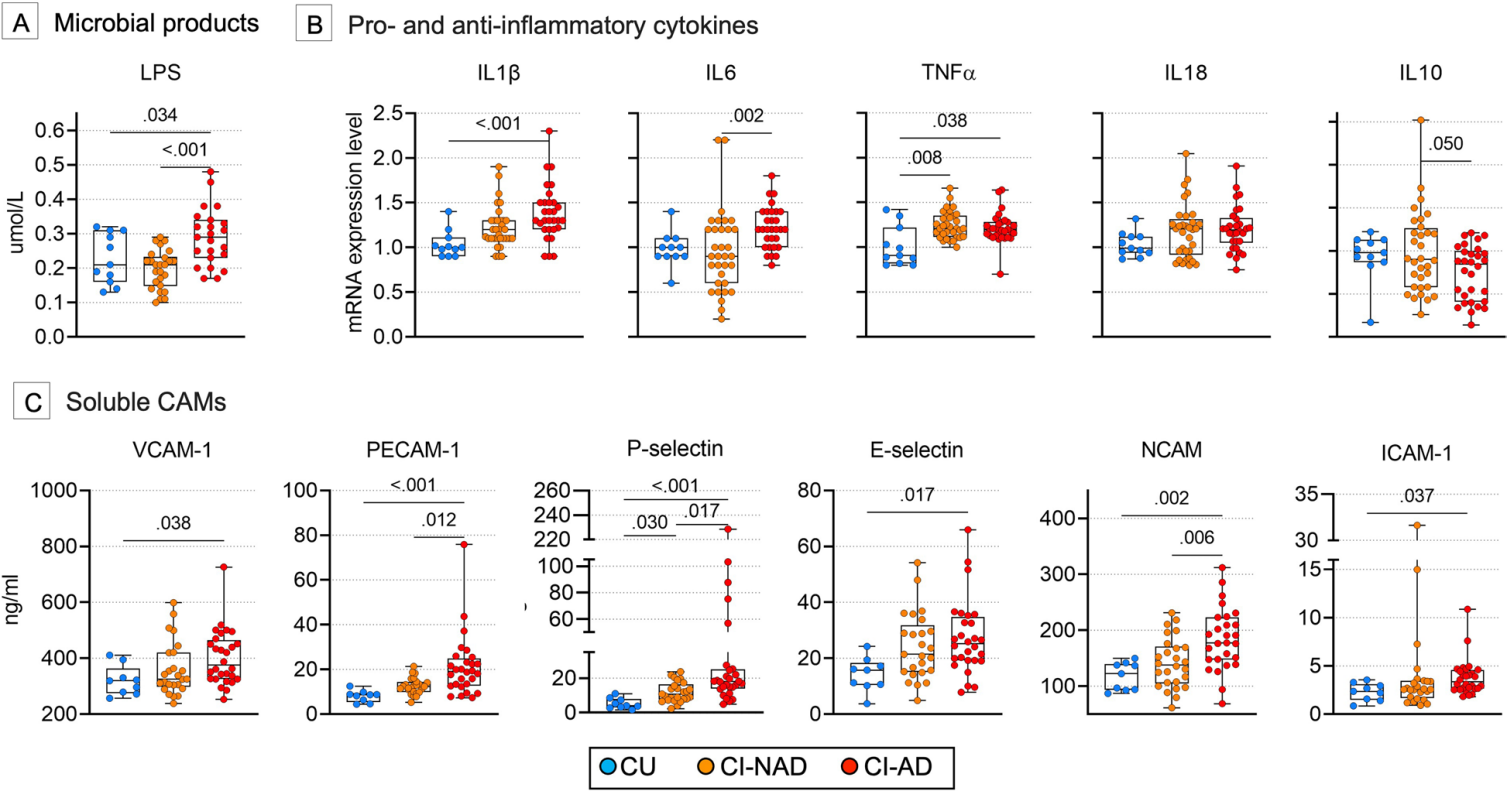
Blood microbiota-gut-brain axis mediators in the blood of study participants. *P*-values were calculated by using one-way ANOVA with Bonferroni’s correction for continuous Gaussian variables (or Kruskall-Wallis test with Dunn’s correction for non-Gaussian variables) for the Gram-negative membrane protein LPS (**A**), the pro (IL1β, IL6, TNFα, IL18) and anti-inflammatory (IL10) cytokines (**B**) as well as the soluble cell adhesion molecules (**C**). Data are presented as box-plots with black horizontal lines indicating medians and circles indicating the subject’s values. Abbreviations: CU, cognitively unimpaired persons; CI-NAD, patients with cognitive impairment not due to AD; CI-AD, patients with cognitive impairment due to AD

Next, we compared the selected putative modulators of the MGBA among groups and we found a specific association of MGBA mediators and cognitive impairment dependent on amyloid status (Fig. 2). CI-AD were characterized by high levels of LPS (*p* < 0.034) (Fig. 2A). Increased expression of pro-inflammatory cytokines was found mainly in CI-AD (IL1β, IL6, TNFα, *p* < 0.038) and to a lesser extent in CI-NAD (TNFα, *p* < 0.038) (Fig. 2B). Moreover, CI-AD showed decreased expression of the anti-inflammatory cytokine IL10 relative to CI-NAD (*p* < 0.050). CAMs analysis showed upregulation of sVCAM-1, sPECAM-1 (reflecting endothelial damage), sP-, sL-Selectin (reflecting vascular changes), sNCAM and sICAM-1 (expressed in response to infections) in CI-AD (*p* < 0.037) and only sP-Selectin (*p* < 0.030) in CI-NAD compared to CU (Fig. 2C).

### Amyloid cascade markers

**Fig. 3:**
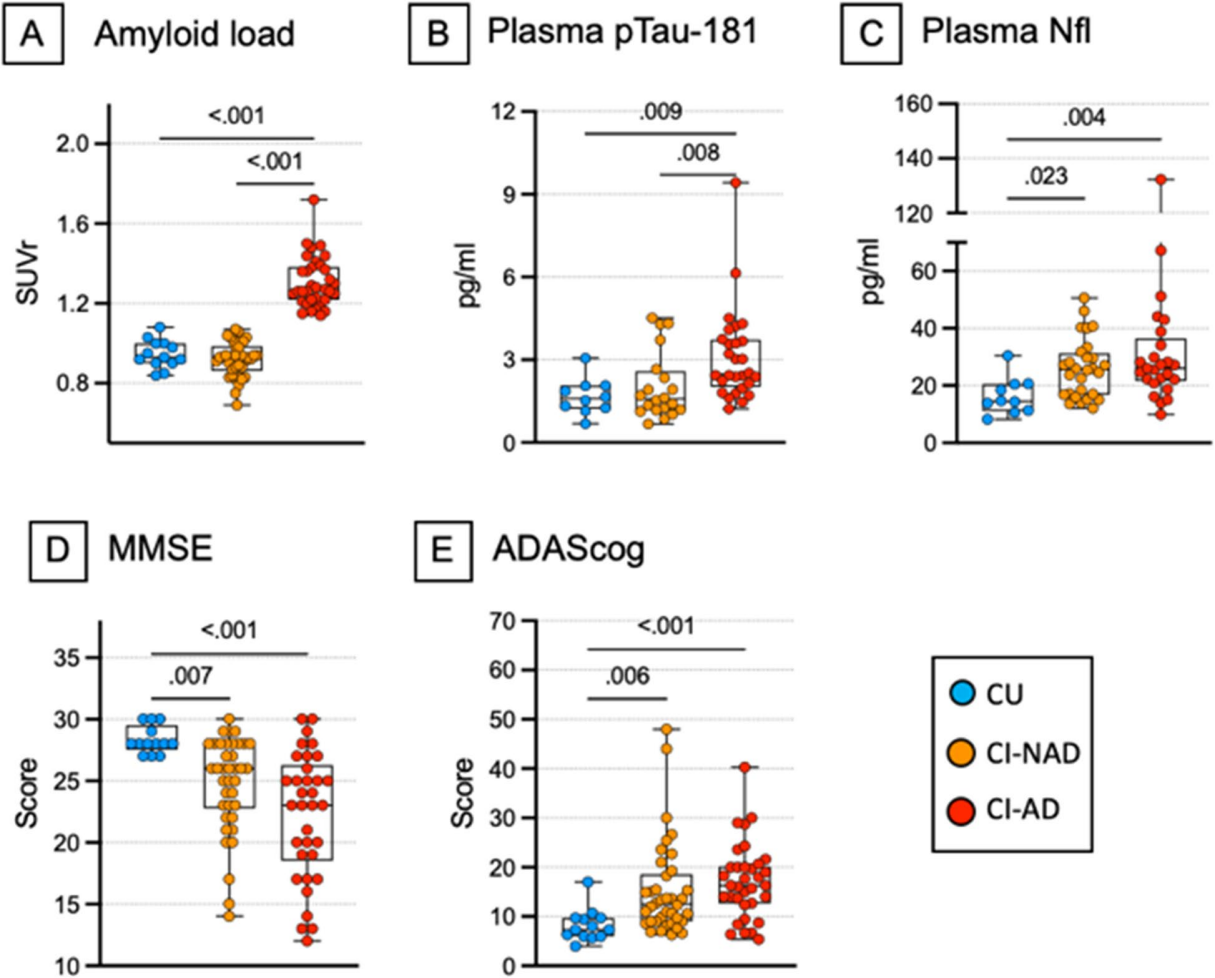
Markers of the amyloid cascade of study participants. *P*-values were calculated by using one-way ANOVA with Bonferroni’s correction for continuous Gaussian variables (or Kruskall-Wallis test with Dunn’s correction for non-Gaussian variables) for the amyloid load (**A**), plasma NfL (**C**) and cognitive measures (**D**–**E**) but not for pTau-181 (**B**), where Mann–Whitney test was applied. Data are presented as box-plots with black horizontal lines indicating medians and circles indicating the subject’s values. Abbreviations: CU, cognitively unimpaired persons; CI-NAD, patients with cognitive impairment not due to AD; CI-AD, patients with cognitive impairment due to AD

In addition to the brain amyloid load measured by PET and used to classify participants into amyloid-positive and amyloid-negative, other markers of the amyloid cascade were measured. Data on plasma pTau-181 showed increased neurofibrillary tangles deposition in CI-AD but not in CI-NAD and CU (*p* < 0.009; Fig. 3B). Increased levels of plasma NfL and worst cognitive performance was found in CI-AD and CI-NAD when compared to CU (*p* < 0.023; Fig. 3C–E).

### Association of microbial genera with MGBA markers and of the latter with amyloid cascade markers

**Fig. 4:**
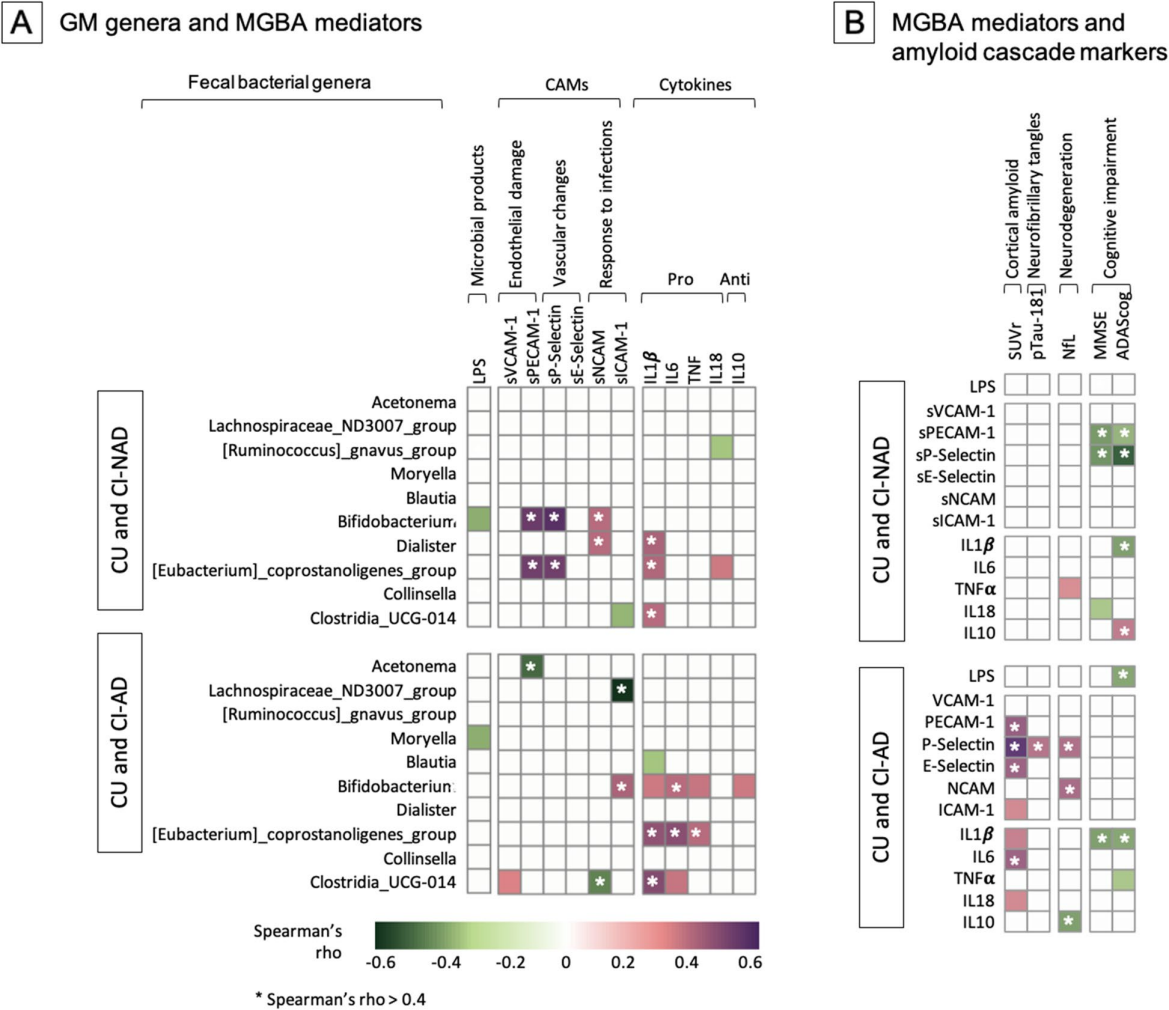
Association matrices of genera with MGBA mediators (**A**) and of the latter with amyloid cascade markers (**B**). Heatmap of the Spearman’s rho coefficient values (pink: positive; green: negative) indicating significant association (age adjusted, *p* < 0.05) in CU and CI-NAD or CU and CI-AD. The star indicates moderate associations (Spearman’s rho value > 0.4). For MMSE and ADAS-cog, higher values reflected higher cognitive scores. Abbreviations: CU, cognitively unimpaired persons; CI-NAD, patients with cognitive impairment not due to AD; CI-AD, patients with cognitive impairment due to AD

In order to test whether AD is associated with distinctive alterations of the GM and MGBA mediator profile, nonparametric association analyses were computed separately in CU and CI-AD or CU and CI-NAD (Fig. 4). Overall, the correlation heatmaps revealed that the genera most abundant in CI groups (i.e., *Bifidobacterium, [Eubacterium] coprostanoligenes group* and *Clostridia_UCG-014*) were associated with endothelial damage, endothelial activation in response to infections and increased expression of pro-inflammatory cytokines (|rho|> 0.35, *p* < 0.044) (Fig. 4A). On the contrary, “protective” genera (i.e., *Acetonema*, *[Ruminococcus]_gnavus_group* and *Blautia*) were associated with endothelial integrity in CI-AD and reduced pro-inflammatory cytokine expression in both groups (|rho|> 0.39, *p* < 0.039).

**Fig. 5:**
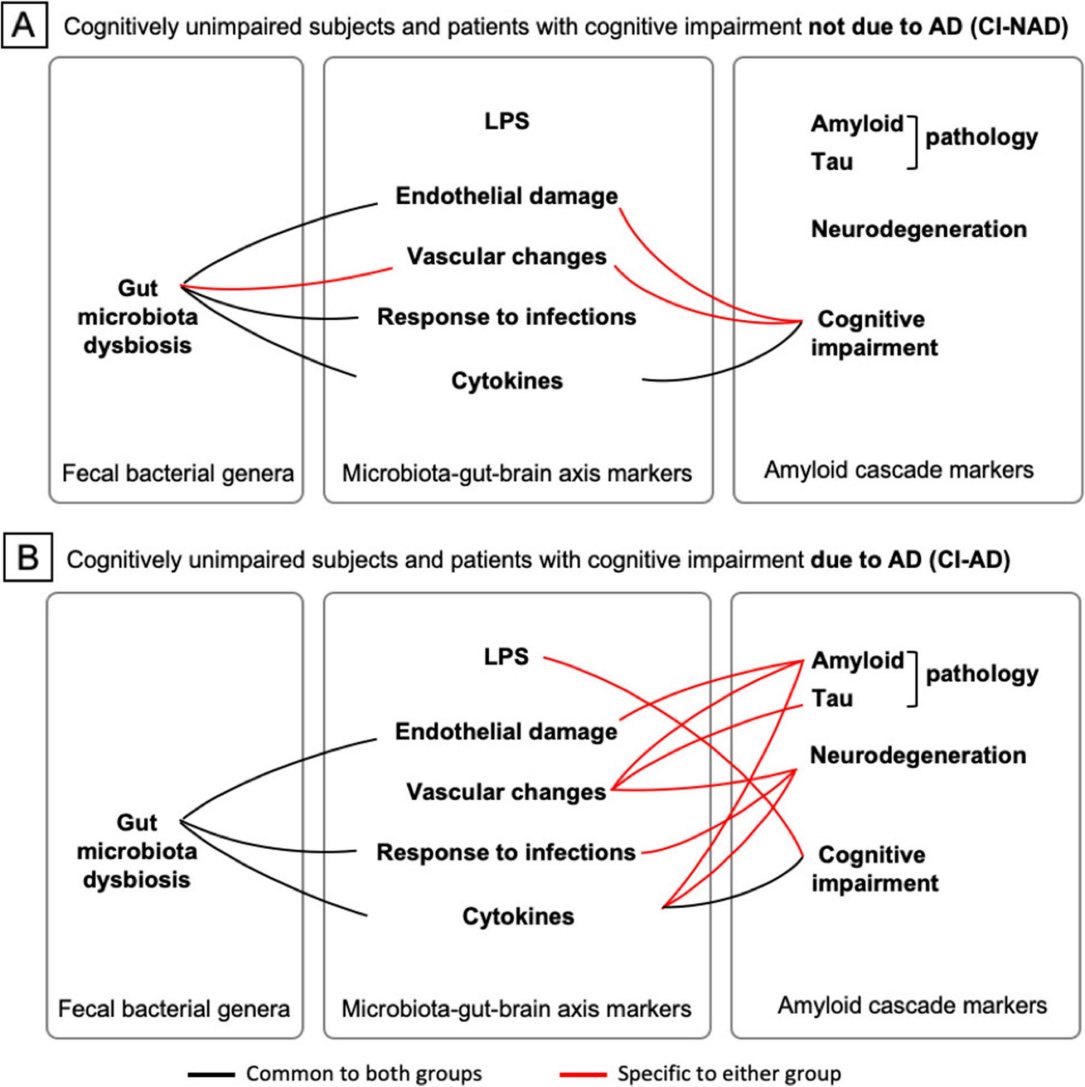
Closest associations (Spearman’s rho value > 0.4) between fecal bacterial genera and microbiota-gut-brain axis mediators and between the latter and amyloid cascade markers in CU and CI-NAD (**A**) and CU and CI-AD (**B**)

The correlation heatmaps of MGBA and amyloid cascade markers showed different patterns of associations in CI-NAD and CI-AD (Fig. 4). In the analysis including CI-NAD, immune and endothelial markers were mainly associated with cognitive decline (|rho|> 0.36, *p* < 0.025), only TNFα was associated with neurodegeneration (rho = 0.37 with NfL, *p* = 0.028) and none with amyloid and tau pathology. In contrast, in the analysis with CI-AD, most of CAMs and cytokines were associated with amyloid and tau pathology and neurodegeneration (|rho|> 0.37, p < 0.025), and only LPS and pro-inflammatory cytokines with CI (|rho|> 0.37, *p* < 0.019). Of note, high levels of sP-selectin (marker of vascular damage) were associated with increased amyloid and tau pathology as well as neurodegeneration (|rho|> 0.44, *p* < 0.012). The different relationship of immune and endothelial MGBA mediators with amyloid cascade markers in CI-AD and CI-NAD was even more pronounced when only moderate associations were considered (Spearman’s rho value above 0.4; Fig. 5).

## Discussion

This study investigated the association of GM with immune and endothelial MGBA mediators and of the latter with markers related to the amyloid cascade in a cohort of cognitively unimpaired persons, patients with cognitive impairment due to AD, and patients with cognitive impairment not due to AD. We report multiple interactions between circulating molecules involved in the MGBA and amyloid aggregation, tau phosphorylation, and neurodegeneration in CI-AD but not in CI-NAD. Our results suggest that peripheral mediators belonging to the MGBA might represent a peripheral signature of AD. Moreover, they strongly support the key role of peripheral mediators in the pathophysiology of AD although the design of the study cannot provide information on the causal chain.

To the best of our knowledge, 10 reports have previously addressed changes in the whole GM in human AD [7–10, 35–40] and MCI patients [9, 10, 35, 38, 39], with often conflicting results. For example, *Blautia* was found both decreased (here and [9, 39]) as well as increased [7, 35] in AD patients compared to controls. Similarly, *Bifidobacterium* was found decreased [7] or increased (here and [35, 40]) depending on the studies. Explanations of these discrepancies can be found in the use of different methods for studying the microbiota profile and to the different criteria for defining Alzheimer’s dementia applied in the studies (Additional file 5). Of the 10 published reports, only 3 reported information on ATN markers [7, 10, 36], and only one used the amyloid positivity finding for the inclusion of patients as in our study [36]. Moreover, sample collection and storage methods, different bioinformatics pipelines, or even the same pipeline on different operating systems have been reported to impact the relative abundance of the dominant bacterial phyla and genera [41, 42]. DNA extraction methods and laboratory locations have been shown to lead to up ten-fold and two-fold differences respectively in the relative abundances of specific bacterial genera, respectively [43].

We confirmed previous human findings demonstrating that blood levels of LPS [44], soluble CAMs (i.e., VCAM-1, PECAM-1, P-Selectin, E-Selectin, NCAM, ICAM-1) [27, 45–49], IL1β, IL6, TNFα [30] are increased in AD patients compared with controls. LPS has been reported to induce amyloid and tau aggregation [50, 51], tau phosphorylation [51], neurodegeneration [52], to reduce synaptic plasticity [53] and to increase microglia density [50] in mouse and rat brains. Our results are consistent with the notion that LPS translocates from the gut to the bloodstream as consequence of increased intestinal permeability [54] and co-localizes with amyloid plaques [55]. The increase in sCAMs reflecting endothelial and vascular damage indicated a general dysfunction of the blood-tissue barriers, including the blood–brain barrier. Furthermore, the upregulation of CAMs involved in the response to infection and controlling leukocyte trafficking is a further indication of widespread vascular inflammation. The latter is thought to promote detrimental processes as it (i) disturbs amyloid-β homeostasis [56] and (ii) facilitates the passage of antigens and bacteria from the gut into the bloodstream [5]. In line with previous findings [57–59], AD patients showed increased pTau-181 and both cognitively impaired groups reported increased NfL and performed worse on the MMSE and ADAScog compared to cognitively normal participants.

### Communication between the compartments

The next step was to integrate the data from the 3 systems and to validate our hypothesis by using correlation analysis. According to the hypothesis of the study, we provide evidence revealing that a specific GM community contributes to AD pathology and cognitive impairment by increasing intestinal permeability and via bacteria products and inflammatory mediators. Although a multitude of animal and in-vitro studies established associations between the immune and endothelial MGBA mediators and AD features [18, 60–67], this relationship in human AD patients is still under-studied. Our analysis revealed that, in patients with cognitive impairment due to AD, (i) sPECAM-1, sP-, sE-Selectins, and IL6 were associated with amyloid and tau pathology; (ii) sP-Selectin, sNCAM, and the decrease of IL10 with neurodegeneration; and (iii) LPS and IL1β with cognitive impairment. Conversely, in patients with cognitive impairment not due to AD, no immune and endothelial MGBA mediators were associated with amyloid and tau pathology and neurodegeneration and many of them were associated with cognitive performance (sPECAM-1, sP-Selectin, IL1β, and decreased of IL10), suggesting that the link between GM alterations and cognitive impairment in non-AD patients likely involved different pathways than AD. While the CI-NAD is a clinically and biologically heterogeneous group, we observed significant associations between GM and MGBA vascular mediators. This, together with the higher occurrence of hypertension within this group, suggests that our CI-NAD might be enriched by patients with vascular disease.

### Limitation

Although this study is one of the few that includes the amyloid marker for subject inclusion, it has also limitations. First of all, this is a small observational study and should be considered with caution due to participant selection and confounding biases. Replication of the results in an independent, larger cohort is needed, which would allow the evaluation of a broader panel of MGBA markers and the use of more complex statistical models. Although our analysis is limited to selected innate immunity and endothelial mediators, gut-brain communication is much more complex and involves, among others, mediators of cellular immunity and circulating molecules directly produced by the gut microbiota such as gut peptides, neurotransmitters, and metabolites. Of note, despite evidence suggesting that the bacterial metabolites’ short-chain fatty acids (SCFAs) have neuroprotective effects, recent studies showed that SCFAs supplementation could increase AD pathology in several AD animal models, possibly through immune cells activation [18, 61, 68]. This suggests that the involvement of SCFAs in microbiota-gut-brain interactions is much more complex than initially thought and that further studies aimed at establishing their relationship are necessary. Furthermore, here we used non-parametric correlations to study the association of markers belonging to the 3 compartments. This, together with the cross-sectional design, prevents us from drawing causal inferences between GM and MGBA marker alterations and AD pathological changes and symptoms onset. Longitudinal studies investigating also the earlier stages of the disease (i.e., amyloid positive and cognitive intact persons) are required to elucidate whether the AD-related microbiota alterations are upstream or downstream to brain AD pathological changes. Last, some of the studied markers were not available for all study participants (Additional file 6).

## Conclusion

This study confirms the presence of a peripheral signature of Alzheimer’s disease featuring microbiota-gut-brain axis markers. The results suggest that the gut microbiota exerts its action on the brain at least in part by modulating endothelial cell function and the levels of circulating inflammatory and microbial products. LPS in the blood and the upregulation of soluble CAMs involved in endothelial damage and vascular changes suggest the presence of a more permeable intestinal barrier in AD. It is therefore plausible that GM products and inflammatory modulators (not only those measured here but also bacteria, fungi, and immune cells) translocate into the bloodstream, reach the brain and trigger the amyloid cascade. The association of LPS and several sCAMs with AD pathology and neurodegeneration provides a new line of evidence for a possible direct link between MGBA and brain pathological changes and offers new biomarkers and treatment targets for AD.

## Supplementary Information

**Additional file 1. **Neuropsychological battery.

**Additional file 2. **DNA amplification, barcoding and sequencing.

**Additional file 3. **Risk factors, drugs and nutritional supplements of study participants.

**Additional file 4. **Beta (A-B) and alpha diversity (C) measures. Beta diversity metrics were computed using normalized data.

**Additional file 5. **Summary of the criteria used to define AD and methods applied to define the GM profile used in the human studies investigating GM alterations in AD.

**Additional file 6. **Number of subjects in the Spearman’s rank correlation analyses of Fig. 5.

## Data Availability

The 16S rRNA sequencing raw data supporting the conclusions of this manuscript are publicly available at https://www.ebi.ac.uk/ena/submit/webin/report/runs/PRJEB55056. The microbiota-gut-brain axis marker dataset analyzed during the current study are available from the corresponding author on reasonable request.

## Abbreviations

ADAScog: Alzheimer's Disease Assessment Scale–Cognitive Subscale
CAM: Cell adhesion molecules
CI-AD: Patients with amyloid-dependent cognitive impairment
CI-NAD: Patients with amyloid-independent cognitive impairment
CU: Cognitively unimpaired persons without brain amyloidosis
LPS: Liposaccharide
MMSE: Mini-Mental State Examination
sE-Selectin: Soluble forms of endothelial leukocyte adhesion molecule
sICAM-1: Soluble forms of intracellular CAM 1
sNCAM: Soluble forms of neural cell adhesion molecule
sP-Selectin: Soluble forms of platelet-activation-dependent granule-external protein
sPECAM-1: Soluble forms of platelet endothelial CAM 1
sVCAM-1: Soluble forms of vascular CAM 1
