# Supplementary material for: A peripheral signature of Alzheimer’s disease featuring microbiota-gut-brain axis markers
Source: Alzheimers Res Ther. 2023 May 31;15:101. doi: 10.1186/s13195-023-01218-5 (PMC10230724; doi:10.1186/s13195-023-01218-5)
Supplement: Supplementary file 1 — Additional file 1. Neuropsychological battery. [file 13195_2023_1218_MOESM1_ESM.docx]

**Additional file 1. Neuropsychological battery.** The cognitive assessment included global cognitive measures (Mini-Mental State Examination (MMSE) and the Alzheimer's Disease Assessment Scale, cognitive portion (ADAS‐Cog)), assessment of memory (logical memory, Rey-Auditory Verbal Learning Test (RAVLT) immediate and delayed recall, recall of The Rey Osterrieth Complex Figure (ROCF)), visuospatial abilities (copy of the ROCF), executive functions (Trial Making Test A and B, Raven’s progressive matrices) and language (Token test, letter and semantic fluencies), behavioral symptoms (Neuropsychiatric Inventory Questionnaire, NPI) and functional level (Basic Activities of Daily Living (BADL) and the Functional Activities Questionnaire (FAQ)).
