# Supplementary material for: A peripheral signature of Alzheimer’s disease featuring microbiota-gut-brain axis markers
Source: Alzheimers Res Ther. 2023 May 31;15:101. doi: 10.1186/s13195-023-01218-5 (PMC10230724; doi:10.1186/s13195-023-01218-5)
Supplement: Supplementary file 2 — Additional file 2. DNA amplification, barcoding and sequencing. [file 13195_2023_1218_MOESM2_ESM.docx]

**Additional file 2. DNA amplification, barcoding and sequencing.** Bacterial DNA was amplified and purified according to 16S Metagenomic Sequencing Library Preparation protocol by Illumina. This protocol allows as a first step to amplify the regions V3 and V4 of the bacterial ribosomal RNA 16S gene, by using the suggested primers (16S Amplicon PCR Forward Primer = 5'TCGTCGGCAGCGTCAGATGTGTATAAGAGACAGCCTACGGGNGGCWGCAG; 16S Amplicon PCR Reverse Primer = 5'GTCTCGTGGGCTCGGAGATGTGTATAAGAGAC AGGACTACHVGGGTATCTAATCC)

and the suggested cycling conditions (3’ at 95°C; 25 cycles: 30’’ at 95°C, 30’’ at 55°C, 30’’ at 72°C; 5’ at 72°C). The resulting amplicon DNA was immediately purified with a magnetic bead step, washed in 80% ethanol and resuspended in Tris-HCl 10 mM. Amplicon DNA integrity was assessed by 2.0% agarose gel electrophoresis on gels containing 0.8 mg/mL ethidium bromide and stored at -20°C. Within one week from storage, amplicon DNA was uniquely dual-indexed, by using the suggested indices (Nextera XT) and the suggested cycling conditions (3’ at 95°C; 8 cycles: 30’’ at 95°C, 30’’ at 55°C, 30’’ at 72°C; 5’ at 72°C). The resulting indexed DNA was immediately purified with a magnetic bead step, washed in 80% ethanol, resuspended in Tris-HCl 10 mM and stored at -20°C. Within one week from storage, indexed DNA was quantified using fluorometric quantification (Qubit, Invitrogen) and the amplicon length was determined by using a Bioanalyzer DNA 1000 chip (Agilent). DNA was then normalized to 4nM, pooled, denatured with NaOH 0.1N, diluted to the final concentration of 10pM and loaded into the MiSeq v3 cartridge (Illumina).
