# Supplementary material for: A peripheral signature of Alzheimer’s disease featuring microbiota-gut-brain axis markers
Source: Alzheimers Res Ther. 2023 May 31;15:101. doi: 10.1186/s13195-023-01218-5 (PMC10230724; doi:10.1186/s13195-023-01218-5)
Supplement: Supplementary file 3 — Additional file 3. Risk factors, drugs and nutritional supplements of study participants. [file 13195_2023_1218_MOESM3_ESM.docx]

**Additional file 3. Risk factors, drugs and nutritional supplements of study participants.**

|  | CU | CI-NAD | CI-AD | P Value |
| --- | --- | --- | --- | --- |
| N | 13 | 38 | 34 |  |
| Risk factors |  |  |  |  |
| Hypertension | 4 (31) | 26 (68) | 16 (47) | **.036**^a^ |
| Diabetes | 2 (15) | 6 (16) | 4 (12) | .878 |
| Vascular disease | 4 (31) | 11 (29) | 4 (12) | .159 |
| Hypercholesterolemia | 7 (54) | 18 (47) | 15 (44) | .835 |
| Stroke | 1 (8) | 6 (16) | 6 (18) | .693 |
| Drugs |  |  |  |  |
| Acetylcholinesterase inhibitors | - | 2 (5) | 3 (9) | .553 |
| Memantine | - | 1 (3) | 0 (0) | .953 |
| Antidepressant/ hypnotic/ anxiolytic | - | 13 (34) | 16 (47) | .267 |
| Antipsychotic | - | 3 (8) | 1 (3) | .916 |
| Nutritional supplements | - | 4 (11) | 7 (21) | .236 |
| Sleep disturbances^b^ | 5.2 (4.8) | 3.9 (4.9) | 3.7 (4.8) | .179 |

^a^ CU vs CI-NAD, p=.017 and CI-AD vs CI-NAD, p=.067.

^b^ The following five night sleep symptoms were evaluated: difficulty falling asleep, waking up frequently in the night, early morning awakenings, not feeling rested in the morning, difficulty staying awake and need to take a nap during the day. For each symptom, respondents were asked to estimate frequency of the symptom during the past month on a five-point scale: 0= never; 1= 1-3 days; 2= 4-7 days; 3= 8-14 days; 4= 15-21 days; 5 = 22-31 days [7].

Figure denotes number (%). Statistical difference between the groups was assessed by Chi-squared test.

Abbreviations: CU: cognitively unimpaired persons; CI-NAD: patients with cognitive impairment not due to AD; CI-AD: patients with cognitive impairment due to AD.
