# Supplementary material for: A peripheral signature of Alzheimer’s disease featuring microbiota-gut-brain axis markers
Source: Alzheimers Res Ther. 2023 May 31;15:101. doi: 10.1186/s13195-023-01218-5 (PMC10230724; doi:10.1186/s13195-023-01218-5)
Supplement: Supplementary file 4 — Additional file 4. Beta (A-B) and alpha diversity (C) measures. Beta diversity metrics were computed using normalized data. [file 13195_2023_1218_MOESM4_ESM.docx]

**Additional file 4. Beta (A-B) and alpha diversity (C) measures.** Beta diversity metrics were computed using normalized data.

**
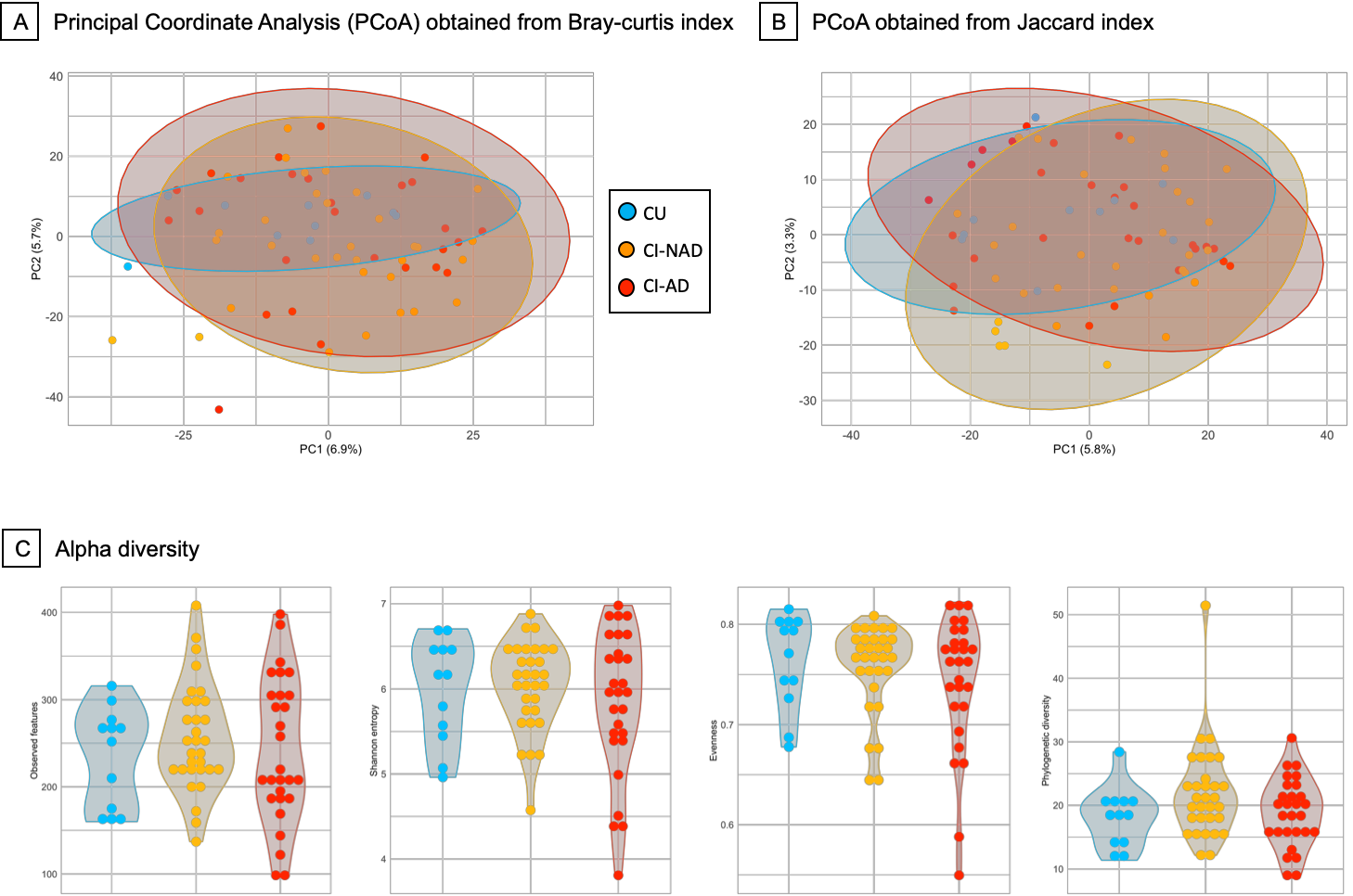
**

Abbreviations: CU: cognitively unimpaired persons; CI-NAD: patients with cognitive impairment not due to AD; CI-AD: patients with cognitive impairment due to AD.
