# Supplementary material for: A peripheral signature of Alzheimer’s disease featuring microbiota-gut-brain axis markers
Source: Alzheimers Res Ther. 2023 May 31;15:101. doi: 10.1186/s13195-023-01218-5 (PMC10230724; doi:10.1186/s13195-023-01218-5)
Supplement: Supplementary file 5 — Additional file 5. Summary of the criteria used to define AD and methods applied to define the GM profile used in the human studies investigating GM alterations in AD. [file 13195_2023_1218_MOESM5_ESM.docx]

**Additional file 5. Summary of the criteria used to define AD and methods applied to define the GM profile used in the human studies investigating GM alterations in AD.**

| **Reference/ Location** | **Participants** | **Criteria** | **GM profile method** | **MMSE of AD and MCI** | **AD biomarkers** |
| --- | --- | --- | --- | --- | --- |
| Verhaar, 2022  Europe | 33 AD, 21 MCI, 116 SCD | NIA-AA | 16S rRNA gene seq, V3-V4 region, ASVs, Silva v138 | AD: 21[19,24]  MCI: 27[25,29]  SCD: 29[28,30] | Not as inclusion criteria |
| Laske, 2022  Australia | 75 A+ AD, 100 CU |  | Shotgun | AD: 22.6±5.1 | Not for CU |
| Guo, 2021  China | 18 AD, 20 MCI, 18 CU | NIA-AA | 16S rRNA gene seq, V3-V4 region, OTU 97%, Greengenes v13.8 | AD: 14.3±6.9  MCI:24.7±2.2 | No |
| Ling, 2021  China | 100 AD, 71 CU | NINCDS-ADRDA | 16S rRNA gene seq, V3-V4 region, OTU 97%, Greengenes v13.8 | AD: 4.3±6.1 | No |
| Li, 2019  China | 30 AD, 30 MCI, 30 CU | NIA-AA | 16S rRNA gene seq, V3-V4 region, OTU, ref database not specified | AD: 18.1±8.2  MCI:27.2±1.1 | No |
| Liu, 2019  China | 33 AD, 32 aMCI, 32 CU | NINCDS-ADRDA | 16S rRNA gene seq, V3-V4 region, OTU 97%, Greengenes v13.8 | AD: 17.9±6.1  MCI:27.3±2.1 | No |
| Haran, 2019  U.S.A. | 24 AD, 33 other dem, 51 CU | Facility medical records | Shotgun | No MMSE available | No |
| Saji, 2019  Japan | 128 MCI (34 dem, 94 nn-dem) | MMSE, CDR | OTUs | dem: 18[15,19]  non-dem: 27[23,29] | No |
| Zhuang, 2018  China | 43 AD, 43 CU | NINCDS-ADRDA | 16S rRNA gene seq, V3-V4 region, OTU 97%, RDP | AD: 14.7±5.0 | No |
| Vogt, 2017  U.S.A. | 25 AD, 25 CU | NIA-AA | 16S rRNA gene seq, V4 region, OTU 97%, Silva, version not specified | No MMSE available | For correlation only |
