# Supplementary material for: A peripheral signature of Alzheimer’s disease featuring microbiota-gut-brain axis markers
Source: Alzheimers Res Ther. 2023 May 31;15:101. doi: 10.1186/s13195-023-01218-5 (PMC10230724; doi:10.1186/s13195-023-01218-5)
Supplement: Supplementary file 6 — Additional file 6. Number of subjects in the Spearman’s rank correlation analyses of Fig. 5. [file 13195_2023_1218_MOESM6_ESM.docx]

**Additional file 6. Number of subjects in the Spearman’s rank correlation analyses of Figure 5.**

| **Panel** | **Maker 1** | **Marker 2** | **N (CU and CI-NAD)** | **N (CU and CI-AD)** |
| --- | --- | --- | --- | --- |
| A | Genera | LPS | 29 | 29 |
|  |  | sCAMs | 28 – 29 (NCAM) | 34 |
|  |  | Cytokines | 35 (IL10) - 36 | 35 |
| B | AmyPET, MMSE, ADAScog | LPS | 37 | 34 |
|  |  | sCAMs | 34 – 35 (NCAM) | 36 (NCAM) - 37 |
|  |  | Cytokines | 43 (IL10) - 44 | 41 |
|  | pTau-181 | LPS | 26 | 29 |
|  |  | sCAMs | 25 – 26 (NCAM) | 34 (NCAM) – 35 |
|  |  | Cytokines | 27 (IL10) - 28 | 34 |
|  | NfL | LPS | 33 | 24 |
|  |  | sCAMs | 33 – 34 (NCAM) | 33 (NCAM) – 34 |
|  |  | Cytokines | 35 (IL10) - 36 | 30 |
